# Factors Associated With Transmission Across Three Waves of SARS‐CoV‐2 in a Prospective Community‐Based Study of Households With School‐Aged Children—Dane County, Wisconsin, 2020–2022

**DOI:** 10.1111/irv.70031

**Published:** 2024-10-30

**Authors:** Ajay K. Sethi, Cristalyne Bell, Derek Norton, Maureen D. Goss, Shari Barlow, Guanhua Chen, Amra Uzicanin, Jonathan L. Temte

**Affiliations:** ^1^ Department of Population Health Sciences University of Wisconsin Madison Wisconsin USA; ^2^ Department of Family Medicine and Community Health University of Wisconsin Madison Wisconsin USA; ^3^ Department of Biostatistics and Medical Informatics University of Wisconsin Madison Wisconsin USA; ^4^ National Center for Emerging and Zoonotic Infectious Diseases Centers for Disease Control and Prevention Atlanta Georgia USA

**Keywords:** genomics, household transmission, respiratory disease surveillance, SARS‐CoV‐2

## Abstract

**Background:**

Household transmission of SARS‐CoV‐2 is a driver of the ongoing COVID‐19 pandemic. Understanding factors that contribute to secondary infection risks (SIRs) can define changing trends and inform public health policies.

**Methods:**

The ORegon CHild Absenteeism due to Respiratory Disease Study (ORCHARDS) prospectively monitors respiratory viruses within the Oregon School District (OSD) in southcentral Wisconsin. Households with students who had ≥ 2 respiratory symptoms were eligible and opted to participate in ORCHARDS. Between October 28, 2020, and May 16, 2022, all household members provided self‐collected nasal specimens on days 0, 7, and 14 for SARS‐CoV‐2 detection using real‐time reverse‐transcription‐polymerase chain reaction. We used logistic regression to investigate individual‐ and household‐level characteristics associated with SARS‐CoV‐2 transmission.

**Results:**

Overall, 127 households comprising 572 individuals (48% female; 52% male; 0.4% nonbinary; 77% ≥ 18 years) had at least one detection of SARS‐CoV‐2. The overall SIR was 47% and decreased over time (pre‐Delta = 72% [95% CI: 58%–83%]; Delta = 51% [40%–63%]; and Omicron = 41% [36%–47%]). Odds of household transmission were 63% lower during the Omicron period compared with the pre‐Delta period (OR = 0.36 [95% CI: 0.13–0.94] *p* = 0.037). Greater household density (members/bedroom) was significantly associated with household transmission during the Omicron period (OR = 6.8, [2.19–21.37] *p* = 0.001). Index case age, illness severity, and individual symptoms were not significantly associated with odds of household transmission.

**Conclusions:**

Greater household density was associated with a higher risk of SARS‐CoV‐2 transmission, but the risk declined over time with subsequent variants. Interplay between variants, prior infection, and individual/household factors may identify modifiable factors (e.g., behavior and vaccination) to reduce future transmission risk.

## 1 Introduction

Across 4 years of the COVID‐19 pandemic, initiated by the ancestral strain of SARS‐CoV‐2, the global community has experienced multiple waves of infections, each attributed to a viral lineage that had a growth advantage over the previously dominant variant. The most researched and discussed variants in the United States have been Alpha, Delta, and Omicron. Despite widespread availability of safe vaccines and specific treatments, both effective in preventing the most severe outcomes, COVID‐19 remained the third leading cause of mortality in the United States through 2022 [1, 2]. Although a sizable proportion of the population has antibodies to SARS‐CoV‐2 acquired from prior infection, vaccination, or both, the future of the pandemic is difficult to predict, with uncertain virulence of future SARS‐CoV‐2 variants [3, 4, 5, 6, 7]. Understanding household transmission dynamics during each wave of the pandemic may provide insight into how trends have changed and what to expect going forward [8, 9].

The risk of contracting SARS‐CoV‐2 is estimated to be 10 times higher within households than in other settings [10]. Previous research has suggested that SARS‐CoV‐2 secondary infection risk (SIR) may be correlated with a variety of factors including age, vaccination status, and variant type. A meta‐analysis of studies, carried out early in the pandemic when the ancestral strain of SARS‐CoV‐2 circulated and restrictions were in place, indicated that 92.3% of secondary cases were among adults [11]. More recent meta‐analyses showed that being fully vaccinated (defined as having completed the primary series of COVID‐19) reduced the likelihood of both contracting SARS‐CoV‐2 and passing the virus on to others in the household, but the magnitude of reduction was greater for Alpha lineage infections compared with Delta or Omicron [9, 12]. To our knowledge, no study to date has examined changing transmission dynamics and SIR within a single population evaluated continuously across multiple waves of the pandemic.

We analyzed data from a prospective, community‐based, and laboratory‐supported household transmission study to determine how SIRs changed over 18 months and which household factors may have been associated with SIR. Our approach was centered on school‐aged children and their households, allowing evaluation of the role of children in household SARS‐CoV‐2 transmission. In addition, whole‐genome sequencing (WGS) data were available for all cases to augment the evaluation of transmission.

## 2 Methods

The ORegon CHild Absenteeism due to Respiratory Disease Study (ORCHARDS) is an ongoing prospective acute respiratory illness (ARI) study among school‐aged children and their household contacts, located within the Oregon School District (OSD, Dane County) in southcentral Wisconsin. This multifaceted community‐based study has been described in detail elsewhere [13, 14]. The study launched at the beginning of the 2014–2015 school year with the primary objective of assessing whether kindergarten through grade 12 (K‐12) school absenteeism could provide an early warning of accelerated transmission of influenza and other respiratory viruses in schools and surrounding communities [14].

Prior to the COVID‐19 pandemic, study coordinators enrolled K‐12 students (index student) with ≥ 2 ARI symptoms and collected specimens from eligible students [13]. In addition, interested households were enrolled and provided materials to self‐collect nasal swab specimens to assess levels of household respiratory pathogen transmission over the course of 7 days. On March 12, 2020, study coordinators ceased in‐person student specimen collections for safety reasons. The study continued uninterrupted by offering households no‐contact supply exchanges and self‐collection of nasal specimens [15] and revealed the first documented case of household SARS‐CoV‐2 transmission in Wisconsin [16].

A protocol change was implemented on October 13, 2020, that expanded the household monitoring period from 7 to 14 days and included both influenza and SARS‐CoV‐2 testing for all household members. On Days 0, 7, and 14, consenting household members self‐collected nasal swabs and recorded on a form any symptoms, symptomatic period, recent travel, likely source of viral contact (if feeling ill), gender, and relationship to the OSD student (Supporting Information). Study coordinators retrieved the specimens and data forms within 24 h of collection and submitted specimens to the Wisconsin State Laboratory of Hygiene (WSLH) for testing. WSLH laboratorians performed real‐time reverse‐transcription polymerase chain reaction (RT‐PCR) tests on specimens using the CDC Influenza SARS‐CoV‐2 Multiplex Assay [17]. Study coordinators notified households of their results, usually within 48 h of specimen collection. In addition, WGS was performed at WSLH on all positive SARS‐CoV‐2 specimens using the Illumina MiSeq platform [18, 19].

To assess household transmission of SARS‐CoV‐2, we analyzed data from the subset of households that had at least one positive RT‐PCR SARS‐CoV‐2 detection during the 14‐day monitoring period. In the study, households could participate multiple times as long as (1) there were at least 7 days between the conclusion of a previous monitoring period and the start of a new monitoring period and (2) the index student had recovered before the onset of new symptoms. Households could be excluded based on the criteria listed in Table 1.

**TABLE 1:** Data elements and definitions used in the assessment of secondary infection risks of SARS‐CoV‐2 within households from data collected in the ORegon Child Absenteeism due to Respiratory Disease Study (ORCHARDS).

Data element	Definition
Variant period	Three time periods conform to pre‐Delta (October 28, 2020, to May 31, 2021), Delta (June 1 to December 31, 2021), and Omicron (January 1, 2022, to May 16, 2022) variant transmission.
Index case	The first case of SARS‐CoV‐2 was detected within the household. When more than one case was detected on the earliest day of detection, the index case was determined using the following considerations in sequential order: (1) earliest self‐reported onset of symptoms, (2) recent travel (more than 50 miles or 1 h away) and self‐reported likely exposure source (classmate, friend, family member, co‐worker, other), and (3) higher cycle threshold (CT) value, under the assumption that higher CT value was more likely indicative of longer duration of infection and earlier acquisition of the virus.
Secondary case(s)	Cases of SARS‐CoV‐2 detection occurred after the initial detection and were possibly linked to the index case.
Acute respiratory infection (ARI)	Participation in ORCHARDS was predicated on a school‐aged child (aged 4–18 years) with an illness characterized by at least 2 of the following 5 symptoms: fever, cough, sore throat, nasal congestion, and rhinorrhea), with symptom onset no more than 7 days prior to time of specimen collection.
Age and gender	We used age in years at the time of specimen collection and self‐reported gender.
Symptoms	Symptoms were self‐reported as yes/no by ORCHARDS participants. Although multiple symptoms are captured in ORCHARDS, we limited this evaluation to fever, cough, rhinorrhea, sore throat, and nasal congestion within the index case.
Severity	Severity in ORCHARDS is self‐reported using a 4‐point scale as follows: 0 = no symptoms; 1 = mild symptoms; 2 = moderate symptoms; 4 = severe symptoms.
Household density	We defined household density as a continuous variable based on the number of household members divided by number of bedrooms.
Vaccination status	We considered a participant vaccinated if they self‐reported receipt of at least one dose of COVID‐19 vaccine prior to participation in ORCHARDS.
Cat ownership	We recontacted households to ascertain cat ownership for a post hoc sensitivity analysis, based on the theoretical risk of transmission based on cats within the household [19].
Concordant/discordant transmission pattern	We assessed whole‐genome sequencing data provided by the Wisconsin State Laboratory of Hygiene to define whether all cases within a household were concordant (within the same lineage) or discordant (more than one lineage present within the 14‐day evaluation period). Please refer to Pango Network for nomenclature rules (https://www.pango.network/the‐pango‐nomenclature‐system/statement‐of‐nomenclature‐rules/). These data were then used in a post hoc sensitivity analysis.
Cumulative participation	The ORCHARDS protocol allows re‐enrollment with subsequent ARI episodes in a K‐12 student. For this study, we considered each enrollment as a potentially new transmission event but assessed the cumulative number of participations, as prior participation was a marker for prior infection. To ensure re‐enrollment was not part of one long continuation of a SARS‐CoV‐2 incidence in the household, re‐enrollment was excluded from analyses if (1) a repeat occurred within 60 days of the prior enrollment and was within the same variant period or (2) a repeat occurred within 30 days and the prior enrollment was in a different variant period [7].

All components of the study were reviewed and approved by the University of Wisconsin Education and Social/Behavioral Science and Health Sciences Institutional Review Boards (protocol 2013‐1357). The study is in full compliance with the Health Insurance Portability and Accountability Act of 1996 (HIPAA), Family Education Rights and Privacy Act (FERPA), and all other federally mandated human subjects regulations. The US Office of Management and Budget approved forms used in this study.

### 2.1 Data Analysis

We performed bivariate comparisons of individual‐ and household‐level characteristics (Table 1) overall and by variant type using Kruskal–Wallis tests for continuous variables and Chi‐squared tests for categorical variables. We defined three calendar periods of interest based on the dominant variant circulating at the time: pre‐Delta (October 28, 2020, to May 31, 2021), Delta (June 1 to December 31, 2021), and Omicron (January 1, 2022, to May 16, 2022). Four families with Omicron detections in late December 2021 (Delta period) were included in the Omicron period.

We estimated overall SIR based on the proportion of nonindex household members who became RT‐PCR positive for SARS‐CoV‐2 within the 14‐day assessment period and used the Clopper–Pearson method for SIR 95% confidence intervals (CI). We also estimated SIR separately during the pre‐Delta, Delta, and Omicron periods.

We used logistic regression to investigate individual‐ and household‐level characteristics associated with household SARS‐CoV‐2 transmission over the period from October 28, 2020, to May 16, 2022. The outcome was the proportion of household participants that contracted SARS‐CoV‐2 infection, excluding the index cases. Index cases were identified first by onset date. In the case of more than one individual with the earliest onset date, several factors were used to determine index designation, including recent travel, known source, and cycle threshold (CT) value as outlined in Table 1. Covariates included variant‐defined calendar period, household density (number of household members/number of bedrooms), index case age, index case severity (none, mild, moderate, or severe symptoms), and the cumulative number of times a household appeared in the dataset (as a proxy for prior infection within the participating household). Appearing more than once meant that there had been a prior SARS‐CoV‐2 detection and monitoring in the household before the most recent occurrence. The associated adjusted SIR is represented by estimating the spread probability for each variant at the mean of the data used to fit the model (i.e., mean values for household density, index age, index severity, and mode of repeat participation).

The number of household nonindex cases was used as a weight in the model to account for variable household sizes. To examine whether covariates differed by period, the same structure—minus prior infection—was individually applied to pre‐Delta, Delta, and Omicron time periods. The model did not include previous household participation because there were no repeating households during the pre‐Delta period. We also assessed individual symptoms in place of symptom severity to see if fever, cough, rhinorrhea, sore throat, or nasal congestion contributed to the increased likelihood of transmission.

We performed two sensitivity analyses to assess result stability with respect to two analysis choices. First, for the two instances when we used the greater RT‐PCR CT value to identify the index case, we instead selected the cases with lower CT values given that CT values in infected individuals are nonmonotonic over time. That is, it is possible that the person with the lower CT value among the two with positive RT‐PCR tests on Day 0 was the one more recently infected. Second, we included a covariate for the proportion of the nonindex members of the household that had been vaccinated. Subjects' vaccination status was determined from their yes/no answer to the question “Did you receive the COVID‐19 vaccine?” Although vaccination is associated with reduced risk of transmission, it was not included in primary analyses because the availability of COVID‐19 vaccines was strongly colinear with variant‐defined calendar periods.

Considering the possible role of cats on household SARS‐CoV‐2 transmission [20], we collected additional data on cat ownership by participating households; a subgroup analysis was performed using a model examining the association between the presence of a household cat and the risk of SARS‐CoV‐2 spread. Because of household pet information being absent for 40 of the 127 (31.4%) households included in the analysis, this covariate was not included in the primary analyses.

We also considered WGS results to assess whether viruses detected within households were entirely concordant (*n* = 114; 89.8%) or discordant (*n* = 13; 10.2%). Again, a subgroup analysis was performed using a model examining the association between the concordance of viruses detected and the risk of SARS‐CoV‐2 spread.

The logistic regression assumption of the dispersion parameter being 1 was checked for all models, and if greater than 1, the estimated dispersion parameter (> 1) was used to adjust standard errors of the covariates in the model before inference was performed, which was the case for all logistic regression models in these analyses.

As supplementary analyses, instead of using the household as independent units of observation and performing logistic regression, we also use generalized estimating equations (GEEs) with a binomial distribution, where the unit of observation is individual (though covariates are still measured at the household level), and account for the within‐household dependency using a same‐household exchangeable correlation structure in the GEE covariance matrix.

Statistical significance was assessed at the 5% level. All analyses were conducted in R version 4.2.0, with *geepack* used for the GEE model fits [21].

## 3 Results

**FIGURE 1:**
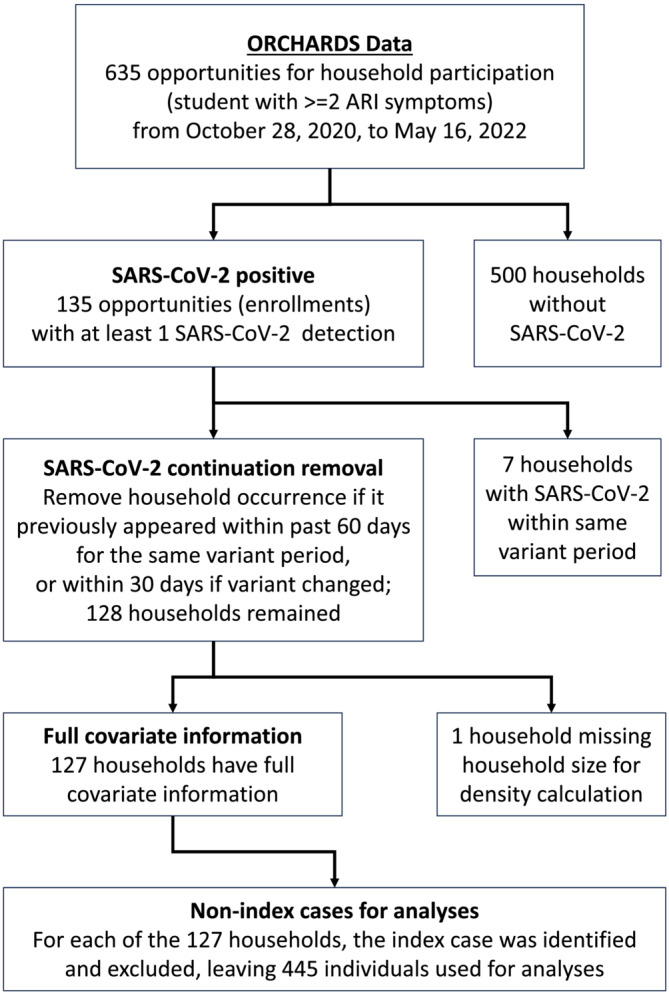
CONSORT diagram of sample selection of households participating in the ORegon CHild Absenteeism due to Respiratory Disease Study (ORCHARDS) and with SARS‐CoV‐2 detections between October 28, 2020, and May 16, 2022.

**FIGURE 2:**
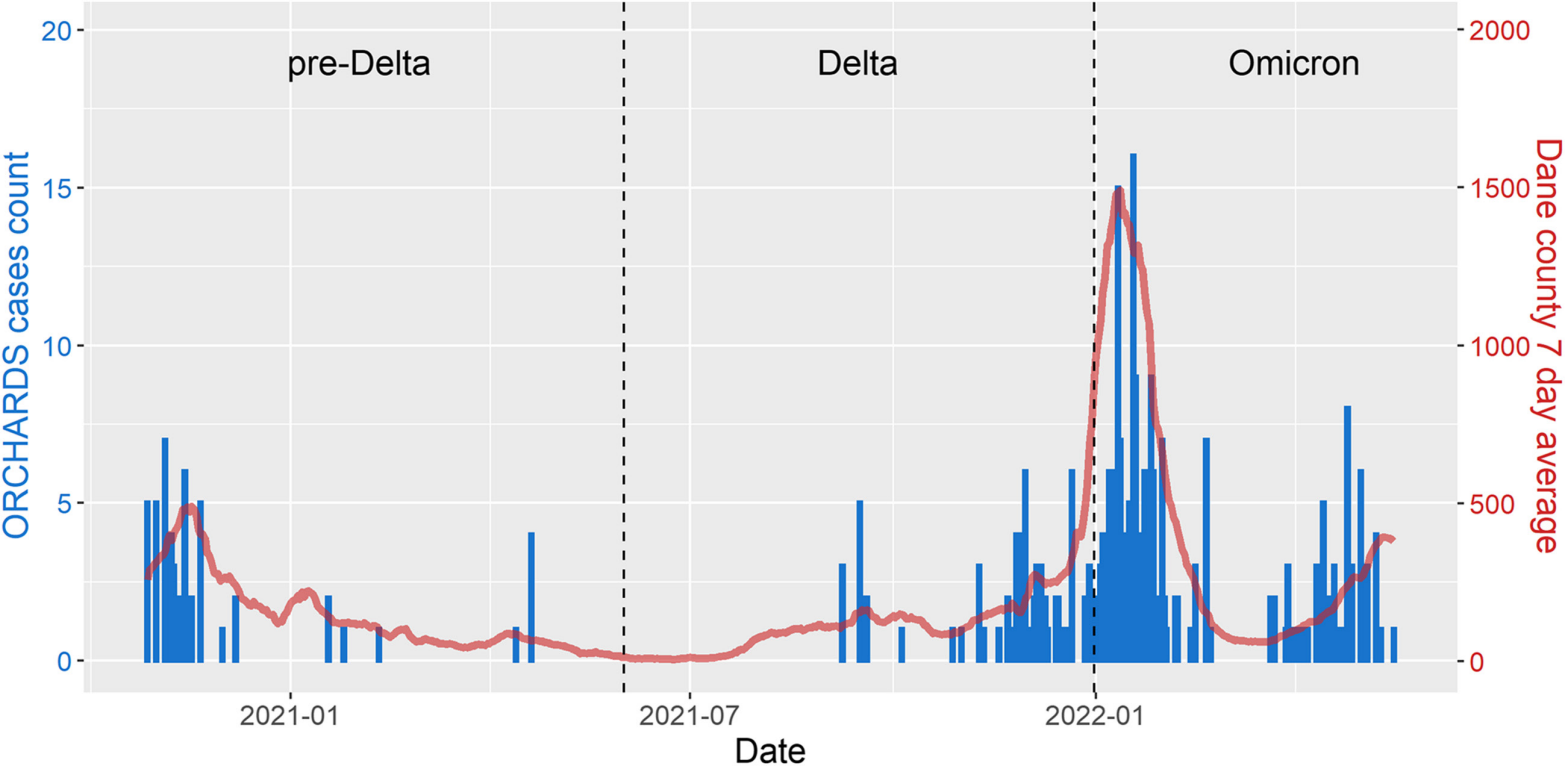
Number of SARS‐CoV‐2 detections within the ORegon CHild Absenteeism due to Respiratory Disease Study (ORCHARDS: blue bars) and within the surrounding county (Dane County, Wisconsin: red line) during October 28, 2020, through May 16, 2021. Vertical dotted lines represent divisions between pre‐Delta (October 28, 2020, to May 31, 2021), Delta (June 1 to December 31, 2021), and Omicron (January 1, 2022, to May 16, 2022) periods used in the study.

Between October 28, 2020, and May 16, 2022, 135 household enrollments occurred with at least one detection of SARS‐CoV‐2 within the household (Figure 1). Nine households participated twice, and one household participated three times. After removal of eight households with early re‐enrollment or missing data, 127 households (94%), representing 572 individuals, remained and had full household participation. Among the 334 SARS‐CoV‐2 detections, 127 (38%) were index cases (Table 2). Most detections (*n* = 219; 65.6%) occurred when Omicron was the dominant variant, with trends consistent with cases reporting at the county level (Figure 2). The vaccination rate within the study population (77.5% by the end of data collection) was higher than that of the United States (68.2%) and Wisconsin (65.5%) but was consistent with county rates (80.1%) [22, 23]. The proportion of household members vaccinated rose from 7% during the pre‐Delta period to 78% during the Omicron period.

**TABLE 2:** Characteristics of individuals and households used for evaluation of household transmission of SARS‐CoV‐2 overall and for variant‐defined calendar periods among ORegon CHild Absenteeism due to Respiratory Disease Study (ORCHARDS) participating households between October 28, 2020, and May 16, 2022.

Individuals' covariates	Individual participants	Individual participants by variant			
		Pre‐Delta	Delta	Omicron	*p*
*n*	572	68	99	405	
Index case, *N* (%)	127 (22.2)	15 (22.1)	23 (23.2)	89 (22.0)	0.964
Variant, *N* (%)					
Pre‐Delta	68 (11.9)				
Delta	99 (17.3)				
Omicron	405 (70.8)				
COVID positive, *N* (%)	334 (58.4)	53 (77.9)	62 (62.6)	219 (54.1)	0.001
Age (mean (SD))	25.04 (17.03)	22.93 (15.67)	24.97 (16.67)	25.42 (17.34)	0.538
Gender, *N* (%)					0.721
Female	264 (48.0)	34 (50.0)	50 (50.5)	180 (47.0)	
Male	284 (51.6)	34 (50.0)	48 (48.5)	202 (52.7)	
Nonbinary	2 (0.4)	0 (0.0)	1 (1.0)	1 (0.3)	
Adult (age ≥ 18), *N* (%)	440 (76.9)	48 (70.6)	79 (79.8)	313 (77.3)	0.363
Relationship (%)					0.989
Student	127 (22.2)	15 (22.1)	23 (23.2)	89 (22.0)	
Mother	126 (22.0)	14 (20.6)	23 (23.2)	89 (22.0)	
Father	116 (20.3)	14 (20.6)	20 (20.2)	82 (20.2)	
Other adult	7 (1.2)	1 (1.5)	0 (0.0)	6 (1.5)	
Sibling	196 (34.3)	24 (35.3)	33 (33.3)	139 (34.3)	
Vaccinated, *N* (%)	388 (67.8)	5 (7.4)	66 (66.7)	317 (78.3)	< 0.001

Families' covariates	Families	Families by variant			
		Pre‐Delta	Delta	Omicron	*p*
*n*	127	15	23	89	
Family spread proportion (mean (SD))	0.45 (0.40)	0.66 (0.39)	0.52 (0.42)	0.40 (0.38)	0.044
Variant (%)					
Pre‐Delta	15 (11.8)				
Delta	23 (18.1)				
Omicron	89 (70.1)				
Family members per bedroom (mean (SD))	1.24 (0.33)	1.35 (0.36)	1.14 (0.38)	1.25 (0.30)	0.132
Proportion of family vaccinated (mean (SD))	0.69 (0.39)	0.07 (0.19)	0.68 (0.35)	0.80 (0.32)	< 0.001
Index age (mean (SD))	21.00 (14.63)	26.07 (15.61)	25.60 (15.97)	18.95 (13.77)	0.053
Index severity, *N* (%)					0.091
Asymptomatic	10 (7.9)	0 (0.0)	1 (4.3)	9 (10.1)	
Mild	40 (31.5)	9 (60.0)	9 (39.1)	22 (24.7)	
Moderate	67 (52.8)	4 (26.7)	12 (52.2)	51 (57.3)	
Severe	10 (7.9)	2 (13.3)	1 (4.3)	7 (7.9)	
Index fever, *N* (%)	62 (48.8)	8 (53.3)	12 (52.2)	42 (47.2)	0.852
Index cough, *N* (%)	95 (74.8)	9 (60.0)	17 (73.9)	69 (77.5)	0.349
Index rhinorrhea, *N* (%)	88 (69.3)	8 (53.3)	16 (69.6)	64 (71.9)	0.353
Index soar throat, *N* (%)	100 (78.7)	11 (73.3)	17 (73.9)	72 (80.9)	0.66
Index nasal congestion, *N* (%)	87 (68.5)	6 (40.0)	13 (56.5)	68 (76.4)	0.008

**FIGURE 3:**
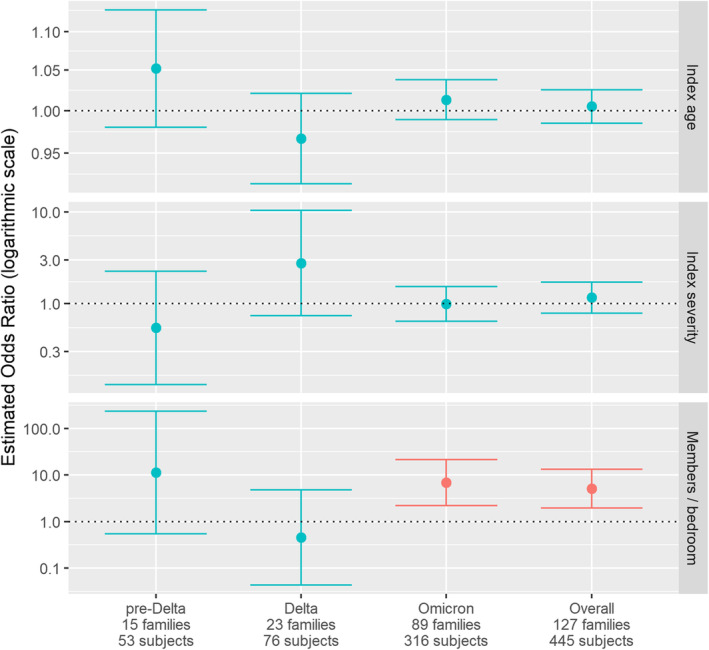
Comparisons of shared regression coefficient results (with 95% confidence intervals) for index case age, self‐reported index case symptom severity (none, mild, moderate, severe), and household density (number of household members per bedroom) for unified (overall) versus stratified models (pre‐Delta, Delta, and Omicron periods). Significant results at *p* < 0.05 are shown in red.

The overall SIR was 47% (95% CI: 42–51), and the estimated risk decreased during each variant‐defined calendar period: pre‐Delta 72% (58–83), Delta 51% (40–63), and Omicron 41% (36–47). There was a statistically significant reduced odds of transmission during the Omicron period as compared with the pre‐Delta period (OR 0.36, 95% CI: 0.13–0.94, *p* = 0.0374). The difference in SIR between the pre‐Delta and Delta periods, and Omicron and Delta periods was not statistically significant (Table 3). Greater household density was associated with increased transmission overall (OR: 5.13 for each increase of one person/bedroom, CI: 1.97–13.35, *p* = 0.0008). In period‐specific models, household density was the only statistically significant covariate and only during the Omicron period (OR: 6.84, CI: 2.19–21.37, *p* = 0.0009). Index case age, illness severity, and individual symptoms were not significantly associated with odds of household transmission (Table 3 and Figure 3). Results from the GEE models (Supporting Information and Figure 1) are consistent with the logistic regression formulation and have very similar point estimates, confidence intervals, and *p*‐values as the logistic regression.

**TABLE 3:** Household‐level factors associated with SARS‐CoV‐2 transmission among ORegon CHild Absenteeism due to Respiratory Disease Study (ORCHARDS) participating households between October 28, 2020, and May 16, 2022. Significant factors are shaded in yellow. When using the Delta period as a reference category in the “Overall” model, the Omicron compared with Delta period statistics are estimate OR = 0.589 (95% CI: 0.34–1.01), *p* = 0.055.

Model	Covariate	Estimated OR	OR 95% CI	*p*	Num. families	Num. subjects	Est. SIR % 95% CI
Overall	Variant: Delta	0.603	0.19–1.89	0.3851	127	445	47 42–51
	Variant: Omicron	0.355	0.13–0.94	0.0374			
	Members/bedroom	5.127	1.97–13.35	0.0008			
	Index severity	1.162	0.79–1.72	0.4521			
	Index age	1.005	0.99–1.03	0.6303			
	Family appearance number	0.346	0.11–1.07	0.0660			
Pre‐Delta	Members/bedroom	11.257	0.54–234.69	0.1182	15	53	72 58–83
	Index severity	0.544	0.13–2.26	0.4014			
	Index age	1.052	0.98–1.13	0.1602			
Delta	Members/bedroom	0.453	0.04–4.78	0.5105	23	76	51 40–63
	Index severity	2.773	0.74–10.38	0.1298			
	Index age	0.967	0.92–1.02	0.2210			
Omicron	Members/bedroom	6.837	2.19–21.37	0.0009	89	316	41 36–47
	Index severity	0.991	0.64–1.53	0.9693			
	Index age	1.013	0.99–1.04	0.2839			

The presence of a household cat was not significantly associated with household SARS‐CoV‐2 transmission risk, though missing information limited this analysis.

The exclusion of 13 households with evidence of discordant detections of SARS‐CoV‐2 resulted in modest reductions in the estimated SIR over the entire study period (47% [95% CI: 42–51] vs. 43% [38–48]), for the Delta period (51% [40–63] vs. 47% [35–59]), and for the Omicron period (41% [36–47] vs 36% [31–42]). Household density continued to be a significant factor in regression models. Of note was a significant difference in self‐reported vaccination proportion between households with concordant (65.2%) and discordant (91.4%) viruses (*p* < 0.001).

## 4 Discussion

In this longitudinal, community‐based study that encompassed periods of pre‐Delta, Delta, and Omicron predominance and included households with school‐aged children who exhibited ARI symptoms, the overall SARS‐CoV‐2 SIR was 47%. The risk for within‐household transmission, however, decreased over the three variant‐defined calendar periods of the COVID‐19 pandemic from an estimated SIR of 72% in the pre‐Delta period, to 51% during the Delta period, to 41% during the Omicron period. Higher household density was the only statistically significant factor associated with SIR, with a six‐fold increase for each increase of one person/bedroom, and only during the Omicron period when two‐thirds of participating households were enrolled in the study and vaccination rates were highest. Index case age was not associated with SIR.

Although vaccination is associated with reduced risk of transmission [9, 12], we were unable to assess its contribution in our primary analyses because the availability and uptake of COVID‐19 vaccines were strongly colinear with the variant‐defined calendar period. Vaccination, albeit predominantly with vaccines based on the original strain, and prior exposure to SARS‐CoV‐2 (OR for prior participation of 0.346 [*p* = 0.066]), may explain why we observed a reduced SIR during the Omicron period, despite this variant being reported as more transmissible than previously dominant variants [9]. Another contributing factor may have been associated directly with study participation. Most households received our laboratory results within 48 h of collection, which may have changed participant behavior (e.g., masking and isolating within the home). Whereas we did not systematically collect data on behavior, we did receive anecdotal information that a positive case resulted in “infection control” activities.

Similar to this evaluation, a prior study across a limited timeframe demonstrated the association of transmission with the number of people per bedroom [24]. Other studies, limited to the early pandemic, discounted the effect of children in household transmission [25, 26]. Although we did not find other statistically significant factors associated with household transmission, prior studies have identified such factors [9, 10, 11, 12]. Neither severity nor individual symptoms influenced SIR in our study, but Frutos et al. found that cough and rhinorrhea increased infectiousness [8]. We specifically assessed cat ownership [20] in post hoc analyses and did not find any significant effect. Of note, we utilized WGS data to refine the estimation of SIR by eliminating households where genetic discordancy between SARS‐CoV‐2 isolated from index cases and secondary cases existed. Removal of households with discordant viruses modestly reduced SIR estimates. Accordingly, studies of household transmission should include evaluation of the genetic signatures of viruses to avoid inflation of transmission risks through misattribution of multiple introductions.

Our findings should be considered in the context of at least five limitations. First, participation in ORCHARDS is predicated on a school‐aged child manifesting ARI symptoms. Because of this, we may under‐accrue adult index cases and bias toward younger age as a factor in transmission. However, we did not find age to be a significant contributor to transmission. Second, limited sample size, especially during the pre‐Delta period, reduced the power of factor assessment. Third, we estimated SIR using the traditional approach of the binomial distribution/logistic regression, which may have resulted in an upward bias [27]. Some investigators have suggested that chain‐binomial or pairwise‐survival models would result in a more accurate measure of household SARS‐CoV‐2 incidence [27]. We elected to use traditional approaches because our primary interest was to estimate the relative effects of individual‐ and household‐level characteristics on household transmission. Additionally, chain‐binomial and pairwise‐survival models require an assumption of transmission times/generations of the virus, which we felt are not firmly established for these SARS‐CoV‐2 variants. Fourth, although ORCHARDS has been a longstanding study, it is representative of a small rural/suburban community and resultant household SARS‐CoV‐2 transmission in southcentral Wisconsin where participants have attained a higher‐than‐average vaccination rate. Accordingly, this may not be reflective of other regions in Wisconsin [22] or throughout the United States [23]. Fifth, as with other studies, we were unable to quantify changes in behaviors resulting from knowledge of infection, nor the frequency and magnitude of behavioral changes, such as mask use and physical distancing, which are associated with mitigating transmission of SARS‐CoV‐2. Accordingly, we were unable to determine whether the decrease in SARS‐CoV‐2 transmission over time was due to vaccination, prior infection, or enhanced use of protective behaviors to reduce secondary infection in households.

Our study had several strengths. First, we followed a single population longitudinally over the multiple pandemic waves allowing examination of SIR through each variant‐dominant wave. Early in the pandemic, as many children attended school remotely, studies suggested that adults were more likely to pass SARS‐CoV‐2 to children than children were to adults, particularly when adults were unable to work remotely [11, 25, 26, 28]. However, when we assessed household transmission dynamics over a longer period of time, we did not observe any effect of age on SIR; this may be consistent with the findings of one meta‐analysis that found that SARS‐CoV‐2 transmissibility among children was higher for later variants [11]. Second, the study framework was well‐established prior to the pandemic, which contributed to high participation and completion rates, and rapid laboratory result turnaround time. Third, our team previously demonstrated the adequacy of self‐collected nasal swab specimens within the study population, which was not a widely used practice prior to the pandemic [15]. Fourth, the number of cases and the variants detected mirrored trends within the surrounding county, which suggests a certain level of generalizability. Fifth, WGS data were available for all SARS‐CoV‐2 detections, allowing for consideration of within‐household concordance, and exclusion of discordant households in post hoc sensitivity analyses.

Limitations of the study include the smaller sample sizes for the pre‐Delta and Delta periods of the data. Additionally, both the logistic regression and GEE formulations of the analyses assume household secondary infections come from the index case only, which is not always true. More complex virus transition models that can account for extra‐household infection risk could be used and may give more precise and less biased estimates [29].

## 5 Conclusion

Over an 18‐month period within the initial 3 years of the COVID‐19 pandemic—and across the periods defined by predominant variants—estimated SIRs declined within participating households with an index SARS‐CoV‐2 infection case. Higher household density was associated with greater SIR overall and during the Omicron period. Moreover, discordance among detected viruses occurred in roughly 10% of households; failure to exclude these households can tend to inflate estimates of SIR.

The risk of SARS‐CoV‐2 household transmission decreased over time over successive periods of variant predominance. Our study demonstrated that household density may play a role in SIR, but more research is needed to understand additional factors that may reduce the risk of transmission, including vaccination coverage, prior infection, and within‐household behavior changes. Widespread immunity, developed after infection or vaccination or both, may eventually drive down levels of community transmission, but it is unclear to what extent this will occur.

## Author Contributions

**Ajay K. Sethi:** writing – review and editing, writing – original draft. **Cristalyne Bell:** writing – original draft. **Derek Norton:** formal analysis, software, writing – review and editing. **Maureen D. Goss:** writing – original draft, writing – review and editing, data curation. **Shari Barlow:** writing – review and editing, project administration, supervision. **Guanhua Chen:** formal analysis, writing – review and editing, software. **Amra Uzicanin:** writing – review and editing. **Jonathan L. Temte:** conceptualization, funding acquisition, methodology, writing – original draft, supervision.

## Disclosure

The findings and conclusions in this study are those of the authors and do not necessarily represent the official position of the Centers for Disease Control and Prevention.

## Ethics Statement

All authors have reviewed and approved the final manuscript. The corresponding author has reviewed the Wiley Author Services *Best Practice Guidelines on Research Integrity and Publishing Ethics* and ensured the manuscript is in compliance with all guidelines.

## Conflicts of Interest

JLT is a member of the Advisory Board for Practice Update Primary Care and has received research grants and in‐kind support from the Quidel Corporation. All other authors report no potential conflicts.

### Peer Review

The peer review history for this article is available at https://www.webofscience.com/api/gateway/wos/peer‐review/10.1111/irv.70031.

## Supporting information


**Table S1** **GEE analysis results** for Household‐level factors associated with SARS‐CoV‐2 transmission among ORegon CHild Absenteeism due to Respiratory Disease Study (ORCHARDS) participating households between October 28, 2020, and May 16, 2022. Significant factors are shaded in yellow. When using the Delta period as a reference category in the “Overall” model, the Omicron compared to Delta period statistics are estimate OR = 0.53 (95% CI: 0.22–1.27), *p* = 0.153.
**Figure S1. GEE analysis results** for comparisons of shared regression coefficient results (with 95% confidence intervals) for index case age, self‐reported index case symptom severity (none, mild, moderate, severe), and household density (number of household members per bedroom) for unified (overall) vs stratified models (pre‐Delta, Delta, and Omicron periods). Significant results at *p* < 0.05 are shown in red.

## Data Availability

The data that support the findings of this study are openly available in the Figshare data repository at https://figshare.com/articles/dataset/Deidentified_datasets_SCV2_HHT_csv/25492831. DOI: 10.6084/m9.figshare.25492831.
