# Supplementary material for: Factors Associated With Transmission Across Three Waves of SARS‐CoV‐2 in a Prospective Community‐Based Study of Households With School‐Aged Children—Dane County, Wisconsin, 2020–2022
Source: Influenza Other Respir Viruses. 2024 Oct 30;18(11):e70031. doi: 10.1111/irv.70031 (PMC11525035; doi:10.1111/irv.70031)
Supplement: Supplementary file 1 — Table S1 GEE analysis results for Household‐level factors associated with SARS‐CoV‐2 transmission among ORegon CHild Absenteeism due to Respiratory Disease Study (ORCHARDS) participating households between October 28, 2020, and May 16, 2022. Significant factors are shaded in yellow. When using the Delta period as a reference category in the “Overall” model, the Omicron compared to Delta period statistics are estimate OR = 0.53 (95% CI: 0.22–1.27), p = 0.153. Figure S1. GEE analysis results for comparisons of shared regression coefficient results (with 95% confidence intervals) for index case age, self‐reported index case symptom severity (none, mild, moderate, severe), and household density (number of household members per bedroom) for unified (overall) vs stratified models (pre‐Delta, Delta, and Omicron periods). Significant results at p < 0.05 are shown in red. [file IRV-18-e70031-s001.docx]

**Supplementary Tables and Figures**

| **model** | **covariate** | **estimated OR** | **OR 95% CI** | **p value** |
| --- | --- | --- | --- | --- |
| Overall | variant: Delta | 0.787 | 0.24 - 2.57 | 0.6910 |
|  | variant: Omicron | 0.417 | 0.16 - 1.09 | 0.0736 |
|  | members / bedroom | 5.991 | 2.16 - 16.63 | 0.0006 |
|  | index severity | 1.173 | 0.79 - 1.74 | 0.4238 |
|  | index age | 1.005 | 0.98 - 1.03 | 0.6433 |
|  | family appearance number | 0.309 | 0.12 - 0.8 | 0.0157 |
| pre-Delta | members / bedroom | 9.813 | 0.49 - 197.97 | 0.1363 |
|  | index severity | 0.485 | 0.16 - 1.47 | 0.2002 |
|  | index age | 1.051 | 0.99 - 1.11 | 0.0834 |
| Delta | members / bedroom | 0.676 | 0.06 - 8.27 | 0.7589 |
|  | index severity | 3.385 | 1.15 - 10 | 0.0273 |
|  | index age | 0.962 | 0.92 - 1.01 | 0.1097 |
| Omicron | members / bedroom | 7.508 | 2.36 - 23.92 | 0.0007 |
|  | index severity | 0.979 | 0.64 - 1.49 | 0.9199 |
|  | index age | 1.016 | 0.99 - 1.04 | 0.2219 |

**Supplemental Table 1: GEE analysis results** for Household-level factors associated with SARS-CoV-2 transmission among ORegon CHild Absenteeism due to Respiratory Disease Study (ORCHARDS) participating households between October 28, 2020, and May 16, 2022. Significant factors are shaded in yellow. When using the Delta period as a reference category in the “Overall” model, the Omicron compared to Delta period statistics are estimate OR = 0.53 (95% CI: 0.22 — 1.27), p = 0.153.


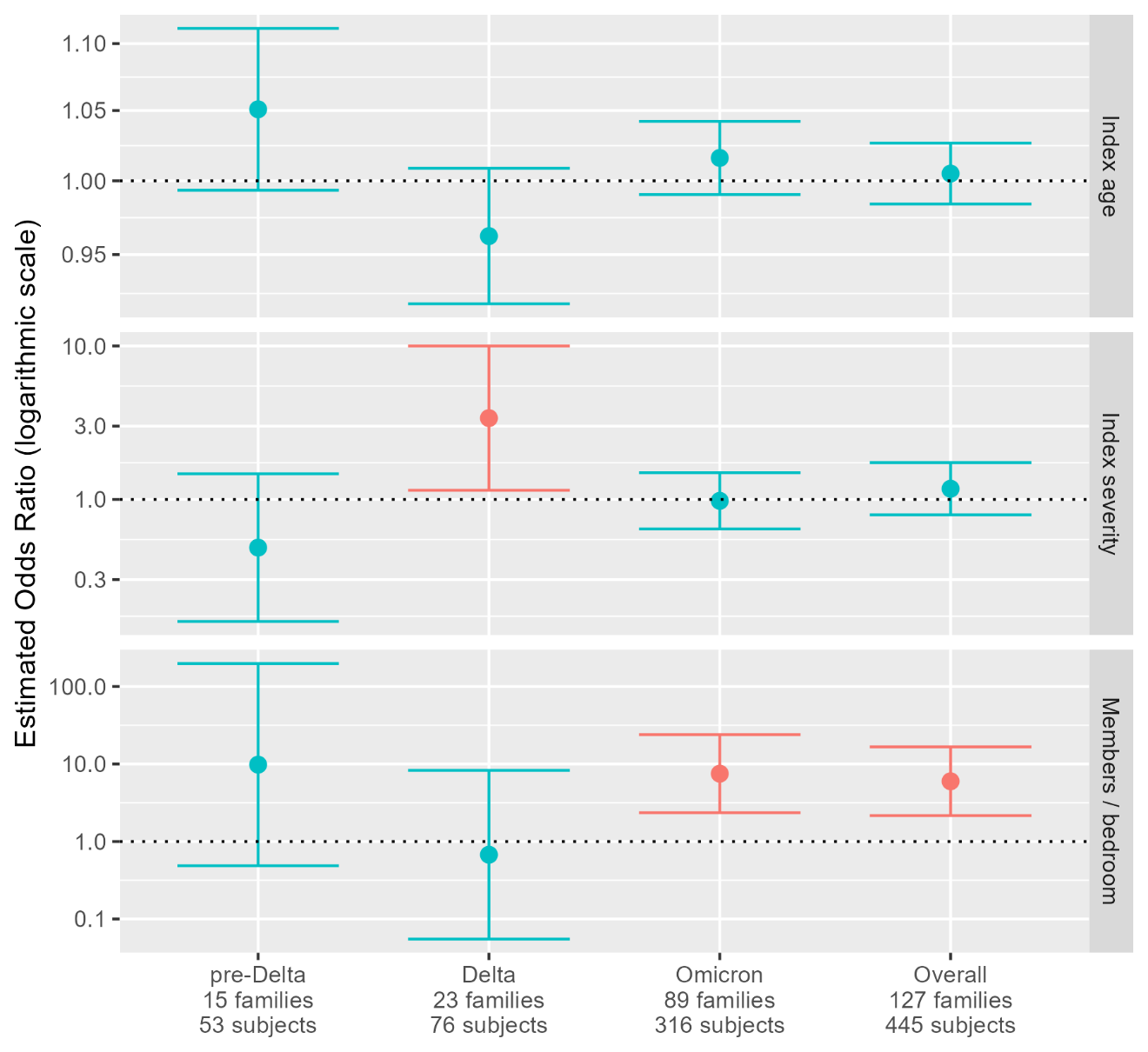


**Supplemental Figure 1. GEE analysis results** for comparisons of shared regression coefficient results (with 95% confidence intervals) for index case age, self-reported index case symptom severity (none, mild, moderate, severe), and household density (number of household members per bedroom) for unified (overall) vs stratified models (pre-Delta, Delta, and Omicron periods). Significant results at P<0.05 are shown in red.
